# Kidney Function in Rice Workers Exposed to Heat and Dehydration in Costa Rica

**DOI:** 10.3390/ijerph19094962

**Published:** 2022-04-19

**Authors:** Jennifer Crowe, Daniel Rojas-Valverde, Marianela Rojas-Garbanzo, Randall Gutiérrez-Vargas, José Alexis Ugalde-Ramírez, José Pablo Ledezma-Rojas, William Cabrera-Alpizar, Minor Salazar-Salazar, Rodrigo Mauricio-La Torre, Lázara Valera-Amador, Berna van Wendel de Joode

**Affiliations:** 1 Central American Institute for Studies on Toxic Substances (IRET), Universidad Nacional, Heredia 40101, Costa Rica; marianela.rojas.garbanzo@una.ac.cr (M.R.-G.); berendina.vanwendel.dejoode@una.ac.cr (B.v.W.d.J.); 2 Centro de Investigación y Diagnóstico en Salud y Deporte (CIDISAD), Escuela de Ciencias del Movimiento Humano y Calidad de Vida (CIEMHCAVI), Universidad Nacional, Heredia 40101, Costa Rica; daniel.rojas.valverde@una.ac.cr (D.R.-V.); randall.gutierrez.vargas@una.ac.cr (R.G.-V.); jose.ugalde.ramirez@una.ac.cr (J.A.U.-R.); william@una.ac.cr (W.C.-A.); 3 Masters Program in Occupational Health, Universidad Nacional-Instituto Tecnológico de Costa Rica, Heredia 40101, Costa Rica; jpabloledezmar@gmail.com; 4 Independent Researcher, San José 10101, Costa Rica; minor18_salazar@hotmail.com (M.S.-S.); rod.ma.lt17@gmail.com (R.M.-L.T.); 5 Medklined Bioanalisis, Nosara 50206, Costa Rica; lazara.valera@uv.es

**Keywords:** agricultural workers, chronic kidney disease, hydration, occupational, heat exposure

## Abstract

The aim of this study was to evaluate heat exposure, dehydration, and kidney function in rice workers over the course of three months, in Guanacaste, Costa Rica. We collected biological and questionnaire data across a three-month-period in male field (n = 27) and other (n = 45) workers from a rice company where chronic kidney disease of unknown origin (CKDu) is endemic. We used stepwise forward regression to determine variables associated with estimated glomerular filtration rate eGFR at enrollment and/or change in eGFR, and Poisson regression to assess associations with incident kidney injury (IKI) over the course of three months. Participants were 20–62 years old (median = 40 in both groups). Dehydration was common (≥37%) in both groups, particularly among other workers at enrollment, but field workers were more exposed to heat and had higher workloads. Low eGFR (<60 mL/min/1.73 m^2^) was more prevalent in field workers at enrollment (19% vs. 4%) and follow-up (26% vs. 7%). Field workers experienced incident kidney injury (IKI) more frequently than other workers: 26% versus 2%, respectively. Age (β = −0.71, 95%CI: −1.1, −0.4), current position as a field worker (β = −2.75, 95%CI: −6.49, 0.99) and past work in construction (β = 3.8, 95%CI: −0.1, 7.6) were included in the multivariate regression model to explain eGFR at enrollment. The multivariate regression model for decreased in eGFR over three month included current field worker (β = −3.9, 95%CI: −8.2, 0.4), current smoking (β= −6.2, 95%CI: −13.7–1.3), dehydration (USG ≥ 1.025) at both visits (β= −3.19, 95%CI: −7.6, 1.2) and pain medication at follow-up (β= −3.2, 95%CI: −8.2, 1.95). Current fieldwork [IR (incidence rate) = 2.2, 95%CI 1.1, 5.8) and being diabetic (IR = 1.8, 95%CI 0.9, 3.6) were associated with IKI. Low eGFR was common in field workers from a rice company in Guanacaste, and being a field worker was a risk factor for IKI, consistent with the hypothesis that occupational heat exposure is a critical risk factor for CKDu in Mesoamerica.

## 1. Introduction

Chronic kidney disease of uncertain etiology (CKDu), also known as chronic kidney disease of non-traditional origin (CKDnt) or Mesoamerican Nephropathy, is a well-documented epidemic in multiple parts of Mesoamerica [1,2,3,4], including the province of Guanacaste in northwestern Costa Rica, where it disproportionally affects men, agricultural workers, manual laborers, and young adults. [5,6] Similar epidemics are documented in other countries, including Sri Lanka [7,8] and India [9]. 

Occupational heat exposure and dehydration are known to be part of the etiology and/or the progression of this disease [2,10], and interventions reducing heat exposure and improving hydration have been shown to have positive effects [11,12]. Most workplace studies conducted thus far have taken place in sugarcane companies [12,13,14,15,16]. However, epidemiological studies have shown that CKDu also occurs in other populations, including cotton, corn and construction workers [17,18], as well as miners and brick makers [18,19,20].

In Costa Rica, sugarcane cutters are at risk for heat illness during the majority of their shift [21,22], motivating the Costa Rican government to institute protections for heat-exposed workers, including serum creatinine (S-Cr) testing in CKDu-endemic areas of the country [23]. The requirement to regularly test for creatinine has made employers and healthcare workers anecdotally aware that abnormally high S-Cr is common in Guanacaste. Nonetheless, aside from sugarcane cutters, virtually no published data exist from other industries in Costa Rica. 

We conducted this research in a rice company for three main reasons. First, rice is grown in the same region as sugarcane, known to be a hotspot for CKDu. Secondly, this workplace has a relatively stable yearlong workforce that includes individuals who have been with the company for many years. Finally, we were able to include participants with different job types in an industrial agricultural setting, allowing for comparisons between groups. The aim of this study was to evaluate heat exposure, dehydration and kidney function in field and other (non-field) workers from a rice company during a three-month period, and to evaluate which variables were associated with kidney function. 

## 2. Materials and Methods

### 2.1. Study Design and Participant Selection

**Figure 1:**
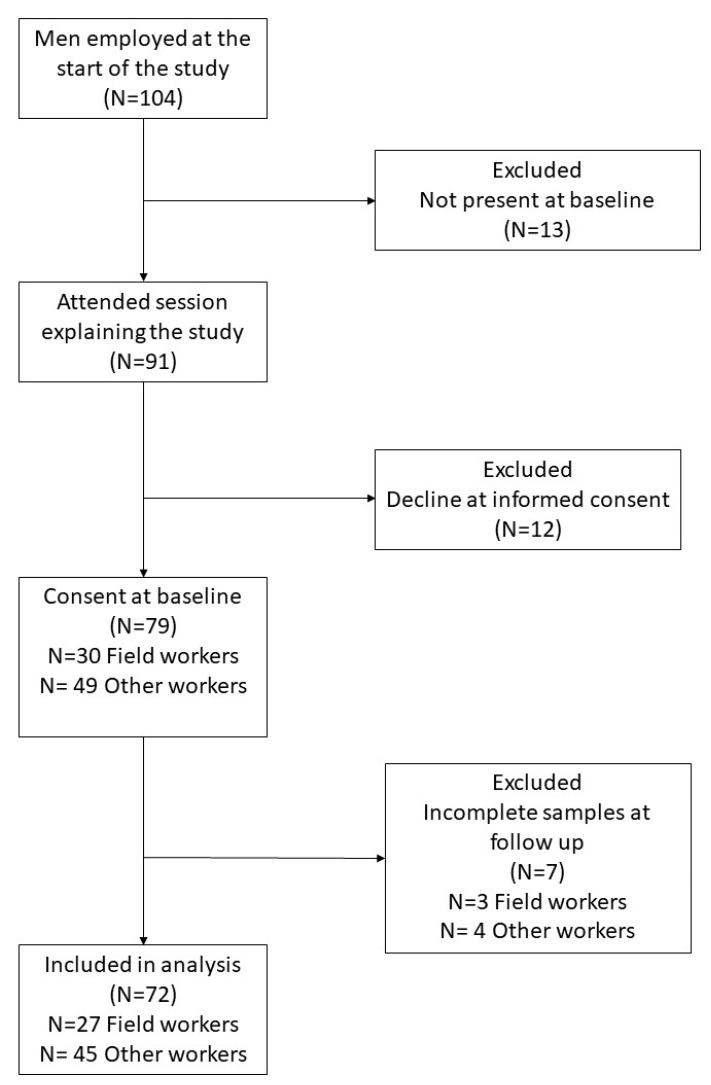
Selection of study participants. Two women were employed, and one provided informed consent, but had incomplete samples at follow-up. Only men are included in the analysis and shown here.

According to company records, 104 men and 2 women were employed at the start of the study. In February 2018, we extended an invitation for an informational session attended by 91 men and 2 women. For the study, 80 men and 1 woman provided informed consent. At the end of the study, the only woman and 8 men (3 field workers and 5 other workers) had incomplete biomarker samples, leaving a total of 72 men in the final analysis (Figure 1) who were demographically similar to the 79 men with baseline samples (Table S1, Supplemental Material).

We collected data for one week each in March, April and May 2018. Trained researchers and assistants applied a questionnaire at baseline to document demographics, prior work, and medical history. We also collected urine and blood samples as well as a symptom questionnaire once per month for each participant. Wet bulb globe temperature (WBGT) was measured each day that biomarker samples were taken (5 days per month), using a QuestTemp monitor [24,25] indoors (below a tin roof with no or partial walls) and outdoors. 

April is excluded from the analysis due to worker holidays during data collection and the delayed delivery of chemical reagents, preventing the analysis of biomarkers. 

This study received funding from the National University of Costa Rica’s Institutional Fund for Academic Development (FIDA) program (communication number CONSACA-009-2015) and was approved by the Institutional Ethical Review Board (CECUNA) of the National University in Costa Rica.

### 2.2. WBGT and Workload

Workload was assessed using Spain´s National Institute of Occupational Safety and Hygiene guideline titled “NTP 323: Determination of metabolic rate” [26]. Workers were observed during the shift by trained field assistants who noted the details of each task as well as the total time each task was performed. In accordance with the NTP 323 “activity component” guideline, we calculated each metabolic rate by summing the resting (basal) rate for the average age of the workers and then calculated the metabolic rate (light, medium or intense) for hands, arms, and trunk according to the guidelines for each task. We then used the guideline to account for speed, distance, weight carried, and inclinations/steps. Each metabolic load was multiplied by the time it was performed before summing all loads. Here, we present the average load per hour and, since workloads varied from day to day, we show results for the highest requirement for each job type. 

We measured wet bulb globe temperature (WBGT) during data collection (5 days each) at baseline and again at follow-up using a QuestTemp 3M^®^ monitor. WBGT measurements were taken wherever participants were working, either outdoors or indoors (tin roof with no or partial walls), but not in air-conditioned offices or vehicles. Finally, we used OSHA recommendations for acclimatized workers to determine whether workers were above recommended exposure limits. 

### 2.3. Biomarker Analysis

Participants were given 100 mL plastic urine cups (NIPRO^®^ Medical Corp., Doral, FL, USA, sterile) and asked to bring a first-morning urine sample on the day that their blood sample was scheduled. All participants were shown how to correctly wash their hands and collect the sample. Samples were stored on ice and taken to a temporary laboratory set up inside the company in an air-conditioned room. A complete urinalysis was conducted within two hours of sample collection using a microscope and reactive strips (CombiScreen 11 SYS., Analyticon) and the color scale provided by the manufacturer. 

Venous blood samples were taken at the workplace by certified professionals using a 5 mL sterile tube (BD Vacutainer^®^, Franklin Lakes, NJ, USA) which contained a spray-coated silica particle activator. Tubes contained a gel polymer to facilitate serum separation. Blood samples were immediately processed by a microbiologist. Samples were centrifuged at 3500 rpm for 15 min for serum extraction and placed in 2 mL Eppendorf containers. All serum samples were frozen at −20 °C and then transported on ice to the university-based laboratory freezer and stored at −20 °C until final analysis. Serum analyses were performed using a biochemical auto-analyzer (Mindray BS-200E, Shenzhen, China) according to the manufacturer’s instructions, and using the original manufacturer´s barcoded reagents. 

### 2.4. Data Analysis

Data were analyzed using Stata version 12 and JMP statistical software from SAS version 8. 

We used the Wilcoxon/Kruskal–Wallis test (rank sums) and Chi-square/Fisher´s exact tests to compare the characteristics of field workers versus other workers. Serum creatinine (S-Cr, mg/dL) was used to estimate glomerular filtration rate (eGFR) using the chronic kidney disease epidemiology collaboration equation (CKD-EPI) [27] with race as “non-black.” USG was analyzed both as a continuous and as a binary variable with values ≥1.025 considered “dehydrated” [28,29]. 

To explore which variables were associated with eGFR at enrollment, we first ran a bivariate linear regression analysis for variables collected in questionnaires on demographics, job history, and medical history, as well as hydration status at enrollment (USG ≥ 1.025) and pesticide use (ever) (paraquat, glyphosate, insecticides and fungicides). We subsequently ran a stepwise forward regression model (probability to enter 0.25, probability to leave 0.10) to determine the multivariate model. We verified the validity of this model by checking for the lack of autocorrelation (Durban–Watson test: 1.5 < d < 2.5), and the distribution of residuals, homoscedasticity, and outliers (Cooks’ distance). As the information on pain and NSAID medication during the last week of enrollment was missing for 11 of the 72 participants (15%), we ran a sensitivity analysis for participants with complete data. 

To evaluate which variables were associated with a change in eGFR over a three-month period, we followed the same procedure as described for eGFR, but for the stepwise forward selecting approach, only considering: age (years), current field worker (yes/no), current alcohol consumption (yes/no), smoking (yes/no), self-reported high blood pressure (yes/no), diabetes (yes/no), obesity (body mass index ≥ 30), dehydrated (USG ≥ 1.025) at enrollment, dehydrated at 3-month visit, dehydrated at both visits, use of pain medication (any), and use of NSAID (yes/no) during the week before the 3-month visit. 

Finally, we calculated an epidemiological measure of incident kidney injury (IKI) across the 3-month period, defined as an increase in serum creatinine ≥0.30 mg/dL or ≥1.5 times the baseline value at enrollment [30]. We used bivariate and multivariate Poisson regression to calculate incidence ratios (IR) of demographical, medical, and occupational variables for IKI over a three-month period. As relatively few persons presented IKI (n = 8), we only included variables with a *p* < 0.05 for bivariate analysis in the multivariate Poisson model. 

### 2.5. Study Population

Characteristics of the study population with complete data are described in Table 1 stratified by field (n = 27) and other (n = 45) workers. The job titles of “other workers” were supervisor, machine operator, hangar worker and mechanic. Supervisors performed indoor and outdoor work, and sometimes traveled by motorcycle or worked in air-conditioned offices. Machine operators drove tractors and other equipment, usually with a roof and often with an air-conditioned cabin. Mechanics usually worked in a large workshop with a tin roof and partially open walls, but at follow-up sometimes traveled to the field where they repaired equipment outdoors. Hangar workers and some machine operators worked with agrochemicals. Workers with agrochemical exposures used appropriate PPE on the days when researchers were present. Only field workers were outdoors for their entire shift. 

Fieldwork varied, including cutting with machete, weeding with a long-handled hoe, opening irrigation ditches using a hoe and, at follow-up, burning fields. Some workers applied agrochemicals by hand on some days and used appropriate PPE when researchers were present. Many field workers used bicycles to move between fields and all work was done on foot. 

All participants were citizens or permanent residents of Costa Rica, but 44% of field workers were born in Nicaragua, compared to 2% of other workers (*p* < 0.0001). Past work in sugarcane fields was reported by 33% of field workers and 4% of others (*p* < 0.05).

Both groups were similar in age, median time in current job, alcohol consumption, current smoking, and obesity. Self-reports of medical conditions diagnosed by a doctor were: hypertension (22%, 20%), diabetes (22%, 9%), and urinary tract infections (11%, 22%). The symptom dysuria (locally “chistate”) was reported by 59% of field workers and 82% of others (*p* = 0.03). Use of pain medication in the previous week was reported by 42% of field and 29% of other workers with percentages for NSAID’s at 13% in both groups, though data were missing for 15% of the participants (Table 1). 

## 3. Results

### 3.1. WBGT and Workload

Field workers had both “moderate” (201–300 kcal/h) and “heavy” (≥301 kcal/h) metabolic loads, whereas other workers generally had “moderate” and sometimes “low” (<201 kcal/h) workloads [24]. Workload was heavier at follow-up due to the end of the harvest and preparation for planting. 

**Figure 2:**
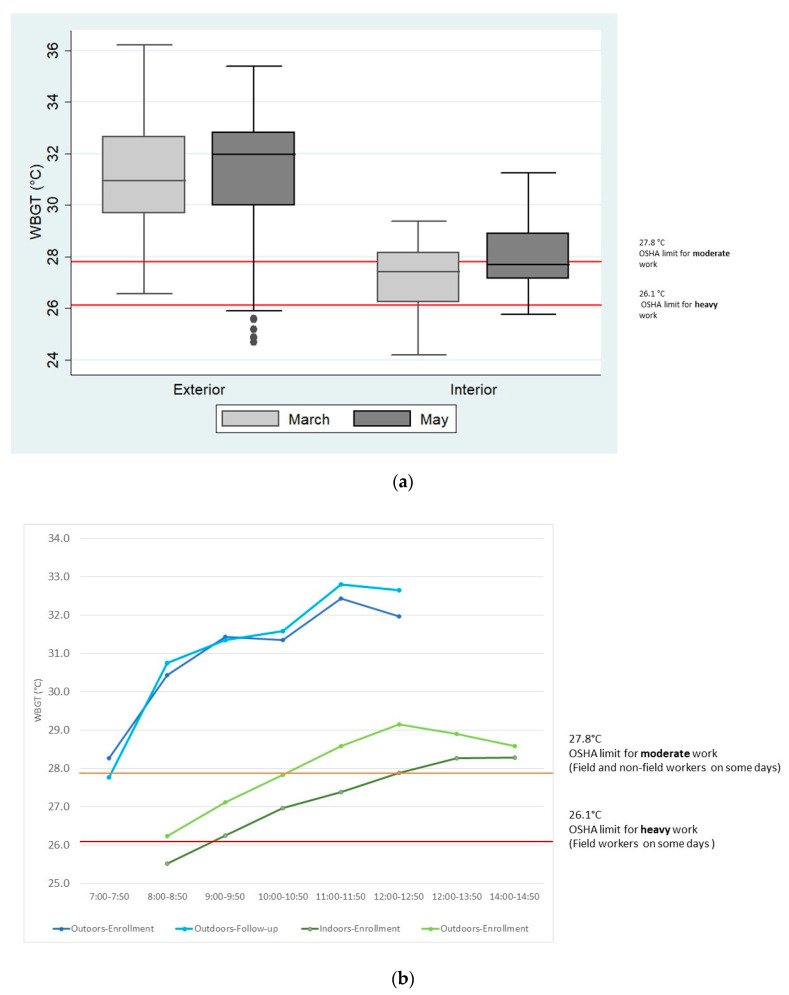
(**a**) WBGT from 7:00 am–12:50 pm during study days in March (enrollment) and May (follow-up) outdoors (usual shift 6:00 am–1:00 pm with workers eating lunch in the field under a roof at approximately noon) and indoors from 8:00 am to 2:50 pm (usual shift 8:00 am–5:00 pm). The Occupational Safety and Health Administration (OSHA) recommended limits for moderate work (28 °C), and heavy work (26 °C) in acclimatized workers is indicated by the red lines. Median WBGT were 30.9 °C and 32.0 °C outdoors in compared to 27.4 °C and 27.7 °C indoors. (**b**) Average WBGT by time in outdoor (blue) and indoor (green) conditions in March (baseline, dark blue and dark green) and May (follow-up, light blue and light green) are shown in figure b. The OSHA recommended limit for moderate work which applied to both field and other workers on some days (27.8 °C), is shown in orange, and the limit for heavy work, which applied to field workers on some days (26.1 °C), is shown in red.

For field workers, shifts were 6:00 a.m.–1:00 p.m., with lunch at noon, usually under a tree or tin roof. Median WBGT between 7:00 a.m. and 12:50 p.m. outdoors in March and May were 31.1 °C and 32.2 °C. Indoor shifts generally lasted from 8:00 a.m.–5:00 p.m., with a lunch break. Median WBGT indoors between 8:00 a.m.–2:50 p.m. were 27.4 °C and 27.7 °C. Maximum WBGT’s in March and May outdoors were 36.2 °C and 35.4 °C compared to 29.4 °C and 31.3 °C indoors. Field workers were above recommended limits for most of the shift during both moderate and heavy work, whereas other workers were above limits for part of the day when they were performing moderate (rather than low) work (Figure 2). 

### 3.2. Urinary Biomarkers

#### 3.2.1. USG

At baseline, dehydration was more prevalent in other workers (64%) compared to field workers (37%, *p* = 0.02), while at follow-up, 44% of workers were dehydrated in both groups (Table 1 and Table 2). Moreover, 26% of field and 40% of other workers were dehydrated in both measures.

#### 3.2.2. Other Urinary Biomarkers

Biomarkers of note include hematuria (traces or higher) in one field worker (4%) and one other worker (2%) at enrollment versus 22% and 9% at follow-up. Proteinuria (≥one plus) in field workers increased from 7% to 30% and decreased in other workers from 27% to 9%. Crystals were present in 15% and 16% at enrollment compared to 7% and 13% respectively at follow-up. Casts were more common in field workers: 11% and 4% at enrollment/4% and 0% at follow-up. No one had casts in both samples (Table 1). 

### 3.3. Serum Biomarkers

High S-Cr (>1.25 mg/dL) was significantly more common in field workers at baseline (41% vs. 11%, *p* = 0.003) and follow-up (41% vs. 18%, *p* = 0.032) (Table 1). Overall, 22% of field and 2% of other workers had high creatinine in both measurements. Mean S-Cr was significantly higher in field workers at follow-up (1.35 vs. 1.12 mg/dL) (Table 2). 

Mean blood urea nitrogen (BUN) was 27.6/27.9 mg/dL at enrollment and 30.9/24.5 mg/dL at follow-up in field and other workers, respectively. The mean BUN-Creatinine ratio was above 20, but there was no significant difference between the two groups: 23.0 vs. 25.1 at enrollment and 23.7 vs. 24.8 at follow-up. More other workers (78%) had a ratio >20 than fieldworkers (59%) at follow-up. The only electrolytes that were significantly different between the two groups were calcium (S-Ca) and magnesium (S-Mg) at enrollment and sodium (S-Na), at follow-up. However, none of the differences were present in both visits and the levels were generally within normal ranges (Table 2).

### 3.4. Kidney Function Outcomes: eGFR at Enrollment, Change in eGFR over Three Months and IKI

Low eGFR (<60 mL/min/1.73 m^2^) was more prevalent in field workers at enrollment (19% vs. 4%) and follow-up (26% vs. 7%) (Table 1). Three field workers (11%) had low eGFR in both months, consistent with clinical criteria for diagnosing CKD; they were 36, 47, and 62 years old. Field workers experienced a median drop of 9.3 compared to 0 mL/min/1.73 m^2^ in other workers (Table 2).

Table S2 shows results from separate bivariate regression models of variables associated with eGFR at enrollment. 

Table 3 shows that the variables age, current field worker, and past work in construction were maintained in the multivariate stepwise regression model of eGFR at baseline, explaining that 20% variability in eGFR [Adjusted R-square (R^2^adj) = 0.20]. Age (β = −0.71, 95%CI: −1.06, −0.3) showed the strongest association, followed by past work in construction (β = 3.75, 95%CI: −0.14, 7.64), while the estimate for current field worker was imprecise (β = −2.75, 95%CI: −6.49, 0.99). The use of pain medication entered into the model, however since pain medication data were missing for 15% of the population at baseline, we conducted a sensitivity analysis for persons who had information about pain medication at enrollment (n = 61). Adding pain medication to the model increased the negative effect for current field worker (β = −4.24, 95%CI:−8.14, −0.33) (Table S3). 

Table S4 shows the results of separate bivariate regression models of variables associated with change in GFR over three months (left hand columns), whilst the results of the multivariate regression model for change in eGFR are presented in Table 4. The variables current fieldwork, current smoking, being dehydrated at both visits, and use of pain medication at 3-month visit were maintained in this model (R^2^adj = 7%). Current fieldwork showed the strongest association: (β = −3.90, 95%CI: −8.19, 0.40), though the result was not statistically significant. 

Finally, incident kidney injury (IKI) across three months was observed in seven field workers (26%) and one other worker (2%) (see Table S4 for results from bivariate analysis). Of these, three (all field workers) had S-Cr above 1.3 mg/dL at baseline. Results from the multivariable Poisson regression model demonstrated an incidence ratio of IKI of 2.22 (95%CI 1.08–5.81) for current field workers and 1.83 (95%CI 0.90–3.60) for workers who reported diabetes (Table 5). 

## 4. Discussion

This study demonstrated that field workers had higher heat exposure and lower eGFR than non-field workers at the same company. They also had a higher risk of IKI. Other biomarkers such as USG were also of note. 

### 4.1. Urinary Biomarkers

USG is a less-than-ideal biomarker for hydration [31], particularly in workers with reduced kidney function, however, we used a very conservative cutoff to determine dehydration (USG ≥ 1.025) [32], and most participants were dehydrated in at least one measurement. Though hydration alone does not guarantee avoidance of kidney damage [30] or heat illness [32,33], studying workplace hydration is crucial [34], and our finding that workers started the shift dehydrated indicates that, despite efforts of this company to comply with Costa Rican regulations [35], improvements must be made.

Hematuria, crystalluria, and casts were more frequent in field workers and, though lower than levels previously reported for sugarcane cutters [36,37], they remain a worrying discovery [19,38,39]. Post-shift samples might give valuable information in this population. We therefore collected post-shift samples in a subset of this population for which analysis is ongoing. 

### 4.2. Serum Biomarkers 

In our study, mean BUN in both groups was above normal, as was the BUN:Creatinine ratio. Other researchers have shown high BUN in sugarcane workers in CKDu-endemic areas [16,19,36], as well as agricultural workers in Florida [40], however, the values in our population were higher than most published values. This may be partially due to the high number of dehydrated participants in our study. 

Some authors have hypothesized that serum sodium and potassium levels may be related to the development of AKI over the course of a work shift [41,42]. In our study, serum sodium was higher in non-field workers at follow-up, while calcium and magnesium were higher (but still normal) in non-field workers at baseline. Though the importance of this is unclear, measuring electrolytes in future studies remains important. 

### 4.3. eGFR, AKI & IKI

In our study, the median eGFR´s were 80.9 and 81.7 mL/min/1.73 m^2^ at baseline versus 75.2 and 79.4 mL/min/1.73 m^2^ at follow-up in field workers and other workers respectively. This is notably lower than reports in Nicaraguan brick makers: 110.6 at baseline and 107.7 mL/min/1.73 m^2^ at follow-up [20]. It is also considerably lower than baseline in El Salvadorian sugarcane cutters: 108 in a coastal population and 119 mL/min/1.73 m^2^ in an inland population [12], however, it is difficult to compare our participants to studies of sugarcane cutters who are usually are pre-screened for S-Cr before hiring [11,14,15]. 

The mean eGFR decrease over three months in field workers (7.6 mL/min/1.73 m^2^) is comparable to other studies in Central America. Wesseling et al. reported mean decreases of 10 and 4 mL/min/1.73 m^2^ in sugarcane cutters and a community referent group over a 9-week period in Nicaragua [36]. In an intervention study with sugarcane cutters in El Salvador, Wegman et al. reported decreases of 5.3 and 3.4 mL/min/1.73 m^2^ in the coastal and inland (intervention) groups, respectively [12]. The metabolic load of rice workers is lower than that of cane cutters, but the cane cutters were required to have a healthy eGFR before being hired. 

We considered an eGFR of <60 mL/min/1.73 m^2^ to be “low.” Though this has become the standard cut-off in epidemiological studies on CKDu, it is admittedly conservative, as it considers stage 2 kidney disease (60–89 mL/min/1.73 m^2^) to be “normal” [27]. To put this into perspective, in a study done on heat-exposed Florida agricultural workers (mostly migrants from Central America and Mexico), eGFR was categorized as “low” below 90 mL/min/1.73 m^2^ [40]. Likewise, a farm worker study demonstrated that only 9% of the males had an eGFR < 90 mL/min/1.73 m^2^, despite being in poorer health than our population [43]. 

Regarding IKI, Laws et al. reported that 4.6% of 284 field workers experienced an increase in S-Cr across the sugarcane harvest of at least 0.3 mg/dL [44]. Similarly, Kupferman and colleagues reported that 10% of 326 Nicaraguan sugarcane workers with normal pre-harvest S-Cr experienced AKI, with nearly half of those workers developing CKD by the end of 12 months [45]. Hansson et al. reported IKI in 7% of 427 sugarcane workers in different job categories, with the highest incidence (27%) in burned cane cutters [30]. Our study showed IKI in 22% of field workers in our study, or 11% when restricting the analysis to workers who had a S-Cr of less than 1.4 (comparable to the Laws et al. study). These percentages are higher than expected, but likely reflect the lack of pre-screening before hiring. Regardless, this finding merits further research in this and other populations.

By taking a second sample at three-month follow-up, we are able to estimate CKD prevalence that would have been quite different had our study used only one measurement: in this case, the prevalence of low eGFR in field workers would have been 19% at enrollment/26% at follow-up (4%/7% in others). Outside of sugarcane workers, few studies have reported on eGFR in CKDu-endemic areas using two measurements over a three-month period. In Nicaraguan brick makers, CKD prevalence using two measures over three months was 14.1% in males [20], a number similar to the 11.1% of field workers in our study. 

### 4.4. Possible Risk Factors

We used three outcome measures for regression models. First, we aimed to explain eGFR at baseline, and our multivariate stepwise regression model included age, status as a field worker, and past work in construction. Secondly, we looked at a decrease in eGFR over a three-month follow-up, where only current status as a fieldworker was associated with decreases in eGFR. Finally, we considered IKI over the course of three months; status as a fieldworker and being diabetic were associated with this outcome. These results are consistent with other investigations that have shown fieldworkers to be at higher risk for these outcomes in sugarcane companies [10], however, this is one of the first studies to demonstrate this phenomenon in other sectors. 

There was no difference in self-reported pesticide use between field and other workers, nor was pesticide use associated with kidney function in our study. This study showed no association between NSAID use and kidney function, however, these data demonstrate that a high number of participants take pain medication, which may be a result of high workloads. 

### 4.5. Strengths and Limitations

The study sample was small; however, it represents most of the workforce at the company. Study participants were aware that we were documenting water consumption, so it is possible that consumption was higher than normal at follow-up. Likewise, days were cut short to allow for data collection, and management may have assigned lighter or shorter tasks than normal. If this occurred, it would have resulted in a lower-than-usual workload, particularly for field workers. Finally, we did not present clothing adjustment factors (CAF) that would decrease the recommended exposure limits for those workers who wore personal protective equipment for part of the shift. Doing so would increase the amount of time that workers (primarily field workers, but also some hanger workers) were exposed on some occasions. 

Ideally, urinary biomarkers should be adjusted for the urinary creatinine concentration to normalize for differences in urinary flow rate. We were not able to do this, which limits the comparability of our data to some studies. Likewise, there are a number of biomarkers that might be useful to study in these populations, however, we were unable to include more biomarkers due to budgetary restraints. 

Including more women remains an important goal for future work, as although most studies show approximately a four-time fold increase in prevalence in men than in women [5,20]. 

We relied on self-reporting for job and medical history, which may have caused some misclassification. In addition, we did not ask about the family history of CKDu, which has been associated with reduced eGFR and CKDu in other studies [20].

To our knowledge, this is the first workplace study in Costa Rica to document heat exposure and biomarkers in a CKDu-endemic region outside of the sugarcane industry. We measured WBGT and estimated metabolic load for each job type instead of using job category as a surrogate, as other studies have done. Because of this, we were able to demonstrate a quantifiable difference in heat exposure between field workers (moderate to heavy) and other workers (light to moderate). Another important strength of the study is the follow-up at three months, allowing us to document sustained low eGFR (as a proxy for CKD prevalence) and IKI over three months. 

## 5. Conclusions

Low eGFR is common in rice workers living in Guanacaste, particularly among field workers. Only current fieldwork was associated with decreased eGFR over three months. IKI occurred in field workers over a three-month period and was associated with current fieldwork and diabetes. These results are consistent with other studies demonstrating that occupational heat exposure is an important risk factor for CKDu in Mesoamerica. Dehydration was also very common in both groups, demonstrating an urgent need to increase efforts to provide adequate water, rest, and shade for heat-exposed workers, in accordance with national regulations. 

## Figures and Tables

**Table 1:** Characteristics at enrollment, unless indicated otherwise, of field and other workers with complete data at 3-month follow-up (n = 72). (Variables differing for field and other workers at *p* ≤ 0.05 shown with asterisk).

Variables	Field Workers (n = 27)			Other Workers (n = 45)
	Median (IQR)			Median (IQR)
Age (years), median (IQR)		42 (30–54)		40 (34–49)
Education (years), median (IQR)		6 (2–7)		8 (6–9)
Current job (years), median (IQR)		9 (4–12)		10 (3–18)
Farmworker (years), median (IQR)		11 (8–20)		0
	n	*%*	n	*%*
Loss-to-follow-up	3	11%	4	9%
Incomplete primary education *	11	41%	4	9%
Born in Costa Rica *	15	56%	44	98%
Married/living as married	20	43%	35	78%
Past work agricultural work at another company	15	56%	29	64%
Past work in sugarcane fields (cutter/irrigation/planter) *	9	33%	2	4%
Past work as a sugarcane cutter *	8	30%	1	2%
Past work in construction	11	41%	12	27%
Consumption of alcohol	19	70%	35	78%
Current smoker	3	11%	3	7%
High blood pressure	6	22%	9	20%
Diabetes	6	22%	4	9%
Urinary tract infection during (ever)	3	11%	10	22%
Chistate (ever) *	16	59%	37	82%
Obese (BMI ≥ 30)	7	26%	13	29%
Pain medication last week at enrollment (a)	10	42%	11	29%
NSAID last week at enrollment (a)	3	13%	5	13%
Pain medication last week at followup	6	22%	9	20%
NSAID last week at followup	1	4%	3	7%
Ever pesticide use (b)	21	84%	27	60%
Ever paraquat use (b)	18	72%	25	56%
Ever glyphosate use (b)	15	60%	19	42%
Ever fungicide use (b, c)	3	12%	8	18%
Dehydrated at (USG ≥ 1.025) enrollment *	10	37%	29	64%
Dehydrated (USG ≥ 1.025) at 3-month follow-up	12	44%	20	44%
Dehydrated at enrollment and 3-month follow-up	7	26%	18	40%
Proteinuria one plus or higher enrollment *	2	7%	12	27%
Proteinuria one plus or higher 3-month follow-up *	8	30%	4	9%
Hematuria (dipstick, traces or more) enrollment	1	4%	1	2%
Hematuria (dipstick, traces or more) at 3-month follow-up	6	22%	4	9%
>3 Erythrocytes (microscopic) (enrollment) (d)	1	5%	0	0%
>3 Erythrocytes (microscopic) at 3-month follow-up	2	7%	1	2%
>5 Leucocytes (microscopic) (enrollement) (d)	0	0%	0	0%
>5 Leucocytes (microscopic) 3-month follow-up	0	0%	0	0%
Casts (any) enrollment (d, e)	3	13%	2	5%
Casts (any) 3 month follow-up (e)	1	4%	0	0%
Crystals (any) enrollment (d, f)	4	18%	8	17%
Crystals (any) 3 month follow-up (f)	2	7%	6	13%
BUN: Creatinine ratio at enrollment >20 at enrollment	17	63%	33	73%
BUN: Creatinine ratio at enrollment >20 at follow-up *	16	59%	35	78%
S-Cr >1.25 mg/dL enrollment *	11	41%	5	11%
S-Cr > 1.25 mg/dL at followup *	11	41%	8	18%
eGFR < 60 enrollment	5	19%	2	4%
eGFR < 60 at 3-month follow-up *	7	26%	3	7%
eGFR < 60 at enrollment and 3-month follow-up *	3	11%	0	0%
eGFR < 90 at enrollment	18	67%	35	78%
eGFR < 90 at 3-month follow-up	24	89%	32	71%
eGFR < 90 at enrollment and 3-month follow-up	16	59%	28	62%
IKI 3 months follow-up *	7	26%	1	2%

Note: * indicates variables differing for field and other workers at *p* ≤ 0.05; (a): Missing information for 3 field and 7 other worker at enrollment, percentages for enrollment are from total non-missing (n = 24 field workers/n = 38 other workers, (b): Missing information for pesticide application for 2 field workers at enrollment, percentages shown are from total non-missing (n = 25 field workers); (c): Carbamate, benomyl or lanate; (d): Missing information for 4 other (non-field) workers at enrollment, percentages shown are from total non-missing (n = 27 field workers/n = 41 other workers); (e): Casts: Field workers at enrollment: 1 erythrocyte & 2 hyaline; Follow-up: 1 hyaline/Other workers at enrollment: 2 erythrocyte/Follow-up: none; (f): Crystals: field workers at enrollment: 4 amorphous urate/Follow-up: 1 uric acid & 1 unspecified; Non-field workers at enrollment: 4 calcium oxalate, 2 amorphous urate & 1 both; Follow-up: 3 calcium oxalate, 1 amorphous urate, 1 triple phosphate, 1 unspecified.

**Table 2:** Urine and serum measurements as continuous variables in field workers (n = 27) and other workers (n = 45) at baseline and follow-up. Significant differences between the mean of field and other workers (*p* ≤ 0.05) are marked with an asterisk.

Variable	Field Workers (n = 27)					Other Workers (n = 45)				
	Mean	SD	Min	p50	Max	Mean	SD	Min	p50	Max
Urine specific gravity (µg/L) at enrollment *	1.017	0.009	1.000	1.015	1.030	1.024	0.008	1.005	1.030	1.030
Urine specific gravity (µg/L) at 3-month visit	1.02	0.007	1.005	1.02	1.030	1.019	0.009	1.005	1.020	1.030
Serum creatinine at enrollment (mg/dL)	1.22	0.308	0.57	1.13	1.96	1.12	0.146	0.85	1.12	1.55
Serum creatinine at 3-month visit (mg/dL) *	1.35	0.392	0.89	1.18	2.37	1.12	0.139	0.85	1.13	1.46
eGFR at enrollment (ml/min/1.73 m^2^)	77.8	21.4	40.7	80.9	128.4	82.6	13.7	51.3	81.7	118.6
eGFR at 3-month visit (ml/min/1.73 m^2^) *	70.2	18.7	33.3	75.2	105.5	82.7	14.0	54.5	80.9	118.6
Difference eGFR (3-month enrollment)	−7.6	23.4	−55.7	−9.3	43.4	0.02	13.5	−25.8	0	43.6
S-glucose at enrollment (mg/dL) (a)	97.22	27.4	31.6	90	252.9	27.43	100.0	69.6	93.6	233.8
S-glucose at at 3-month visit (mg/dL) (a)	134.03	86.7	70.7	104.4	450.4	111.68	37.5	80	102.8	331.7
S-urea N (BUN) at enrollment (mg/dL)	27.63	9.5	5.5	25.6	41.5	27.91	7.0	14.9	27.3	45.7
S-urea N (BUN) at 3-month visit (mg/dL)	30.94	11.6	4.9	29.1	59.1	27.47	5.6	16.7	27.8	38.3
BUN: Creatinine ratio at enrollment (mg/dL)	23.0	6.7	5.3	22.8	37.6	25.1	6.6	11.5	24.4	43.9
BUN: Creatinine ratio at 3-month visit (mg/dL)	23.7	7.9	2.6	23.1	37.9	24.8	5.8	12.1	25.3	36.8
S-uric acid at enrollment (mg/dL)	5.84	1.8	1.33	6.01	8.81	5.67	1.1	3.16	5.45	8.61
S-uric acid at 3-month visit (mg/dL)	6.29	1.4	4.19	6.01	9.73	5.73	1.2	3.84	5.47	8.92
S-albumin at enrollment (g/dL)	4.31	1.0	1.67	4.42	5.99	4.48	0.2	4.05	4.46	4.92
S-albumin at 3-month visit (g/dL)	4.48	0.3	3.63	4.5	4.88	4.53	0.2	3.97	4.57	4.95
S-CPK at enrollment (Ul/L)	213.48	174.9	18	176	893	160.09	71.2	43	138	343
S-CPK at 3-month visit (Ul/L)	216.78	192.9	62	150	672	196.43	116.3	62	159.5	400
S-Na at enrollment (mmol/L)	141.08	7.8	104.7	142.8	149.6	143.50	4.4	137.6	142.1	153.5
S-Na at 3-month visit (mmol/L) *	139.05	4.2	130.85	139.58	147.93	143.84	4.0	134.39	144.39	149.9
S-K at enrollment (mmol/L)	3.96	0.5	2.84	3.9	5.05	4.11	0.4	2.96	4.06	5.01
S-K at 3-month visit (mmol/L)	4.05	0.4	3.16	4.1	4.75	4.37	0.4	3.15	4.46	4.95
S-Cl at enrollment (mmol/L)	98.28	3.1	92.52	98.5	106	99.59	3.8	91.24	100.17	107.7
S-Cl at 3-month visit (mmol/L)	97.26	3.9	88.32	97.66	102.82	98.51	2.6	91.74	98.92	104.61
S-Ca at enrollment (mmol/L) *	2.16	0.2	1.24	2.22	2.41	2.26	0.1	2.04	2.25	2.52
S-Ca at 3-month visit (mmol/L)	2.27	0.1	2.12	2.25	2.48	2.30	0.1	2.06	2.3	2.58
S-Mg at enrollment (mmol/L) *	0.77	0.1	0.44	0.79	0.94	0.92	0.1	0.61	0.94	1.12
S-Mg at 3-month visit (mmol/L)	0.81	0.1	0.51	0.8	1.07	0.82	0.1	0.58	0.81	1.28

(a) Not all of the samples were fasting samples due to workers’ schedules, so results should be interpreted with caution.

**Table 3:** Multivariate stepwise regression model for eGFR at enrollment. The variables age, current field worker, and past work in construction were maintained in the model, including all participants with complete data at follow-up (n = 72), explaining 20% of variability in eGFR [Adjusted R-square (R^2^adj) = 0.20].

Y	Term	Estimate	*p*	Lower 95%	Upper 95%
eGFR at enrollment	Intercept	110.69	<0.00	95.61	125.76
	Age (41 ± 10)	−0.71	<0.00	−1.06	−0.36
	Current field worker (38%)	−2.75	0.15	−6.49	0.99
	Past work in construction (32%)	3.75	0.06	−0.14	7.64

**Table 4:** Multivariate regression model for change in eGFR (Month 3–baseline). The variables current fieldwork, current smoking, being dehydrated at both visits, and use of pain medication at 3-month visit were maintained in this model (R^2^adj = 0.07), including all workers (n = 72).

Term	β Estimate Difference in eGFR Month 3-Baseline	*p*	Lower 95%	Upper 95%
Intercept	−11.78	0.01	−19.93	−3.62
Current field worker (38%)	−3.9	0.07	−8.19	0.4
Current smoker (8%)	−6.2	0.1	−13.72	1.31
Dehydrated at both visits (USG ≥ 1.025) (35%)	−3.19	0.15	−7.58	1.2
Pain medication at follow up (21%) (a)	−3.15	0.22	−8.24	1.95

(a) pain medication during the past week reported at follow-up.

**Table 5:** Poisson regression for IKI (yes or no) over three months (variables differing for field and other workers at *p* ≤ 0.05 shown with asterisk).

Variables	Exp(β) (=IR Estimate, 95%CI)			*p*
(Intercept)	0.14	0.00	0.28	<0.001
Diabetes	1.83	0.90	3.60	0.08
Current field worker *	2.22	1.08	5.81	0.05

## Data Availability

The datasets generated during and/or analyzed during the current study are available for the corresponding author upon reasonable request.

## Supplementary Materials

The following supporting information can be downloaded at: https://www.mdpi.com/article/10.3390/ijerph19094962/s1, Table S1: Characteristics of study population (men only) at baseline (n = 79) and at with complete data at three month follow-up (n = 72); Table S2: Results from bivariate linear regression analysis (n = 72) presenting beta estimates with 95%CI for selected variables.; Table S3: Sensitivity analysis for multivariate stepwise regression model for eGFR at baseline (Table 3) excluding cases where pain medication data were missing at baseline (15% of participants). (R2adj = 0.27); Table S4: Bivariate analysis for (1) difference in eGFR after three months and (2) IKI from Poisson regression.
