# Supplementary material for: Kidney Function in Rice Workers Exposed to Heat and Dehydration in Costa Rica
Source: Int J Environ Res Public Health. 2022 Apr 19;19(9):4962. doi: 10.3390/ijerph19094962 (PMC9100597; doi:10.3390/ijerph19094962)
Supplement: Supplementary file 1 [file ijerph-19-04962-s001.zip › ijerph-1655694-supplementary.pdf]

Table S1. Characteristics of study population (men only) at baseline (n=79) and at with complete data at three month follow-up (n=72).

	All men (n=79)	Complete data at follow-up (n=72)
	median (IQR)	median (IQR)
Age (years), median (IQR)	40 (33-49)	40 (33.4-49.5)
Education (years), median (IQR)	6 (6-9)	6.5 (6-9)
Current job (years), median (IQR)	10 (3-16)	10 (3-15.5)
Farmworker (years), median (IQR)	9.5 (4-12)	9 (4-12)
	%	%
Incomplete primary education	20%	21%
Born in Costa Rica	84%	82%
Married/living as married	77%	76%
Past work similar work at another company	62%	61%
Past work in sugarcane	34%	32%
Past work as a sugarcane fieldworker	15%	15%
Past work not in agriculture	32%	32%
Consumption of alcohol	75%	75%
Current smoker	9%	8%
High blood pressure	20%	21%
Diabetes	14%	14%
Urinary tract infection during (ever)	20%	18%
Chistate (ever)	75%	74%
Obese (BMI ≥30)	25%	28%
Pain medication last week(a)	33%	34%
NSAID last week (a)	22%	13%
Ever pesticide use(b)	69%	69%
Ever paraquat use (b)	62%	61%
Ever glyphosate use(b)	48%	49%
Ever fungicide use (b, c)	15%	16%
Dehydrated at (USG ≥1.025) enrollment	54%	54%
Proteinuria one plus or higher enrollment	19%	19%
Hematuria (dipstick, traces or more) enrollment	4%	3%
Casts (any) enrollment (d)	8%	7%
Crystals (any) enrollment (d, f)	16%	6%
eGFR<60 enrollment	10%	10%
eGFR<90 at enrollment	71%	74%

a) 15 missing data for first column/ 10 missing for second column, percentage shown out of complete data

b) 8 missing data for first column/ 2 missing for second column, percentage shown out of complete data

d) 4 missing data in both columns, percentage shown out of complete data

Table S2. Results from bivariate linear regression analysis (n=72) presenting beta estimates with 95%CI for selected variables (estimates with $p<0.05$ in bold).

Variables			β estimate for eGFR at enrollment		
	median	IQR	estimate	95%CI	
Age (years)			**-0.69**	**-1.05**	**-0.34**
Current job (years)			-0.48	-0.97	0.00
	n	%	estimate	95%CI	

Field worker	27	38%	-4.89	-14.20	4.42
Past work at another company	44	61%	2.01	-6.27	10.29
Past work as a sugarcane cutter	9	13%	-3.53	-9.56	2.51
Past work in sugarcane fields	11	15%	-3.40	-8.94	2.14
Past work in construction/mining/fishing	23	32%	2.80	1.47	7.07
Consumption of alcohol	54	75%	-2.40	-12.17	7.36
Current smoker	6	8%	4.90	-20.08	29.88
High blood pressure	15	21%	-3.14	-14.75	8.48
Diabetes	10	14%	-3.92	-16.36	8.51
Ever urinary tract infection	13	18%	1.70	-8.71	12.11
Ever chistate	53	74%	1.13	-7.58	9.84
Obese (BMI ≥30)	20	28%	3.72	-4.97	12.41
Pain medication last week(a)	21	34%	9.27	-0.57	19.11
NSAID last week (a)	8	13%	-0.48	-17.87	16.90
Ever pesticide use	48	67%	-2.92	-11.20	5.36
Ever paraquat use	43	60%	-0.71	-8.74	7.32
Ever glyphosate use	34	47%	-1.88	-10.18	6.42
Ever carbamate, lannate, benomyl use	8	11%	-0.65	-13.45	12.16

(a)n= 3 (11%) of farmworkers and n=8 (16%) of other workers with missing data at baseline; Percentages shown are out of complete data.

Table S3. Sensitivity analysis for multivariate stepwise regression model for eGFR at baseline (Table 3) excluding cases where pain medication data were missing at baseline (15% of participants). (R2adj = 0.27).

Y	*Term*	*Estimate*	*p*	*Lower 95%*	*Upper 95%*
eGFR at enrollment	Intercept	112.14	0.00	96.65	127.64
	Age	-0.72	0.00	-1.08	-0.35
	Current field worker	-4.24	0.03	-8.14	-0.33
	Past work in construction	3.21	0.16	-1.26	7.68
	Pain medication during the last week*	4.11	0.05	-0.06	8.29

*Pain medication variable excluded for 15% with missing medication data at baseline.

Table S4. Bivariate analysis for 1) difference in eGFR after three months and 2) IKI from Poisson regression. .

**Variables**	**median**	**IQR**	**Dif-eGFR estimate**	**95%CI**		**IKI estimate**	**95%CI**	
Age (years)			0.15	-0.26	0.57	1.01	0.95	1.08
	**n**	**%**	**estimate**	**95%CI**		**estimate**	**95%CI**	
Field worker	27	38%	-7.59	-17.58	2.41	**2.44**	**1.20**	**6.30**
Consumption of alcohol	54	75%	1.96	-9.66	13.58	0.84	0.43	1.83
Current smoker	6	8%	**-13.07**	**-25.52**	**-0.61**	1.79	0.69	3.63
High blood pressure	15	21%	0.94	-7.54	9.42	1.01	0.39	2.05
Diabetes	10	14%	-8.23	-18.24	1.77	**2.12**	**1.06**	**4.14**
Obese (BMI ≥30)	20	28%	0.00	-7.34	7.35	0.58	0.13	1.34
Pain medication last week (3-month visit) (a)	21	34%	**-9.98**	**-19.62**	**-0.35**	1.58	0.81	3.19
NSAID last week (3-month visit) (a)	8	13%	-7.09	-17.72	3.54	1.40	0.54	2.86
Current pesticide use	14	19%	3.24	-10.94	17.42	1.16	0.17	4.81
Current paraquat use	12	17%	-3.54	-16.96	9.88	1.41	0.21	5.81
Current glyphosate use	10	14%	7.83	-11.28	26.94	0.76	0.04	4.16
Dehydrated at enrollment	39	54%	0.35	-8.56	9.26	0.50	0.19	1.02
Dehydrated at 3-month follow-up	32	44%	-5.47	-13.55	2.62	1.12	0.54	2.30
Dehydrated at enrollment and 3-month follow-up	25	35%	-3.96	-11.94	4.03	0.74	0.29	1.51

(a) 3 (8%) of farmworkers and 7 (16%) of non-farmworkers with missing data at baseline; Percentages shown are out of complete data.
